# Sex differences in the association between internalizing symptoms and hair cortisol level among 10-12 year-old adolescents in China

**DOI:** 10.1371/journal.pone.0192901

**Published:** 2018-03-09

**Authors:** Qingyun Lu, Fada Pan, Lingling Ren, Jing Xiao, Fangbiao Tao

**Affiliations:** 1 Department of Maternal, Child and Adolescent Health, School of Public Health, Anhui Medical University, Hefei, Anhui, China; 2 Department of Child and Adolescent Health, School of Public Health, Nantong University, Nantong, Jiangsu, China; 3 Colleage of education science, Nantong University, Nantong, Jiangsu, China; Erasmus MC, NETHERLANDS

## Abstract

Although numerous studies have described the relationship between HPA axis dysregulation and internalizing symptoms among adolescents, research using hair cortisol concentrations in pre- and young adolescent samples has not been reported. We investigated the association of self-reported internalizing symptoms with cortisol concertration in hair among pre- and young adolescents aged 10–12 years. Forty-six boys and 39 girls supplied a hair sample of at least 3 cm in length for an analysis of this period (3 months) cortisol excretion. Saliva cortisol reactivity to the Trier Social Stress Test for Children (TSST-C) also was assessed. The study found a positive association between ratings of depressive symptoms and cumulative levels of hair cortisol only in boys. Furthermore, higher ratings of anxiety symptoms were associated with lower hair cortisol concertration and lower saliva cortisol reactivity among girls. This study provides the first evidence for the notion that depressive symptoms in boys are associated with long-term cortisol concertration in hair, whereas anxiety symptoms in girls are associated with HPA-axis hypoactivity, when hair cortisol concentrations and saliva cortisol reactivity to acute stress are assessed concurrently.

## Introduction

Internalizing symptoms in children and adolescents, such as non-clinical depression and anxiety traits, are key health issues inchildhood and adolescence. Brady and Kendall [1],who performed a literature review of internalizing disorders in children and adolescents, estimated that 15.9–61.9% of children and adolescents have anxiety and depression disorders. Early emerging internalizing symptoms predict later internalizing disorders [2]. The hypothalamic–pituitary–adrenal (HPA) axis, which is acentral component of the body’s neuroendocrine response to stress, is thought to play a central role in the pathophysiology of depressive and anxiety disorders.

Indeed, dysregulation of the HPA-axis has been found to be related to anxiety and depressive (internalizing) symptoms, not only in adults [3,4], but also in children and adolescents [4,5]. However, it has been increasingly recognized that associations between cortisol reactivity and internalizing problems are weaker and less consistent in older childhood and early puberty. The study of HPA-axis function has mainly been performed by measurement of cortisol levels or activity. Previous research typically has found a positive pattern of association between depression and acute cortisol reactivity under the standardized stress task [6,7] or basal cortisol level or dynamics during mid- to late adolescence [8,9]. The pattern of association involving the assessment of depressive symptoms in children, in contrast, is inconsistent: no association between depressive symptoms and cortisol reactivity [10,11] or a negative association has been observed between depressive symptoms and acute cortisol reactivity [12–14]. Anxiety, which is another form of symptom internalization, is reported to be mostly some studies have observed no association between anxiety and cortisol reactivity in related to hypo-HPA axis activity in children and adolescents [10,12,15]. Yet, some studies have observed no association between anxiety and baseline cortisol level in older children and young adolescents [16], or a positive association between anxiety and acute cortisol reactivity [11–13].

Furthermore, sex differences appear to modify the relationship between HPA-axis activity and internalizing problems in children and adolescents [17,18]. Sex differences in depressive and anxiety disorders have been reported to emerge during adolescence, with girls having higher rates of both depressive and anxiety symptoms than boys [19–21]. The possibility that the HPA axis is maximally sensitive to experiences during adolescence has implications for psychopathology, especially for females, which may imply an early pathway leading to the preponderance of depression and anxiety that emerges in females in adolescence [22]. Moreover, some research provides evidence of the developmental interface of sex differences in the association between HPA-axis functioning and depression in pre- and young adolescence, and indicates that sex differences in depressive symptoms or disorders are generally not apparent until adolescence [14].

Previous research mostly has relied on salivary or plasma cortisol measurements, with HPA activity being assessed by acute cortisol reactivity to a stress test (e.g., physical cold, CRH infusion, or social situations), or for baseline cortisol collection after waking up in the morning, or a 24-hour cortisol secretion patterns. However, these measures may fall short when documenting the long-term status of the HPA-axis. Hair cortisol has been hypothesized to provide a retrospective maker of cumulative activation of the HPA-axis. This is because the level of cortisol in hair reflects exposure to stress over time, with each segment of hair growth reflecting prior HPA-axis activity [23]. Notably, the measurement of cortisol in hair has also emerged as a promising strategy for assessing psychosocial stress [24]. Indeed, a number of studies have explored the association between levels of hair cortisol and internalizing symptoms among adults [24,25–28], however, such study of hair cortisol levels has not been reported in pre- and young adolescents sample.

Numerous human studies have found sex differences in HPA-axis regulation appearing at puberty [9,29–31], that adolescent girls have higher free saliva cortisol levels [32], the upregulation of cortisol activity across the day [33], and the cortisol response upon awakening is higher in girls than boys [34]. Therefore, we expected there would be sex differences in hair cortisol among young adolescents. This notion has been supported by non-human research. Lower levels of hair cortisol in male vervet monkeys have been reported to emerge at puberty [35]. To the best of our knowledge, previous studies of psychological problems did not examine sex differences in cortisol concentrations from hair among in pre- and young adolescents, or sex differences in the link between hair cortisol reactivity and internalizing symptoms. Thus, the purpose of the present study was to obtain information about sex differences in hair cortisol under stable conditions in pre- and young adolescents (ages 10 to 12 years), and to obtain a better insight in the association between cortisol measures and internalizing symptoms in this population. In addition, saliva samples to measure changes in cortisol levels following the Trier Social Stress Test (TSST) were obtained to compare different methodologies of cortisol assessment.

## Materials and methods

### Participants and procedure

This study is part of an ongoing longitudinal study of urban adolescents in Xuzhou City, China. The purpose of this cohort study was to assess the internalizing symptoms and cognitive behavior in pre- and young adolescents.The participants with significant medical or psychological problems were excluded from participation via a screening procedure administered by trained study personnel at the recruitment stage. Ninety-four healthy paricipants (50% girls) were recruited from interested 7th grade students at two urban public schools. Three subjects were excluded due to the exclusion criteria. Three subjects were excluded due to the exclusion criteria (one had psychotropic drug history, and two had systemic corticosteroid used during the past three months). Six girls at their menstrual period were also excluded. We did not considered them because the hormone in the menstrual period may impact current cortisol secretion. Completed data from 46 boys and 39 girls were analyzed (mean age = 11.4 ±0.3 years). Anthropometric data including height and weight were collected by well-trained interviewers when the participants visited the experimental room. The self-administered questionnaires requested pubertal stage, socioeconomic status information, and information on internalizing symptoms are described further. Then the individual start to take part in the Trier Social Stress Test for Children (TSST-C). Saliva samples to measure changes in cortisol levels following the were obtained. Each participant was tested individually. When the individual completed the TSST-C and saliva collection, hair samples were taken from the posterior vertex of the scalp. The study protocol was approved by the Medical Ethical Committee of Anhui Medical University, and written consent was obtained from all participants and their parents. All participants received a small monetary reward of ¥20.

## Measures

### Anthropometric measures

Height was measured to the nearest 0.1 cm using a metal column height measuring stand and weight was measured with a precision of 0.1 kg using calibrated, sensitive scales. BMI (kg/m^2^) was calculated as body weight (kg) divided by height (m) squared. Pubertal stage was self-assessed by Tanner criterion of genital stage for boys and breast stage for girls using realistic images. Parental education levels (Junior high school or less, Senior high school, College or higher) and family income levels (low, middile, high) were self-reported by adolescents.

### Hair cortisol measurement

Hair samples of approximately 150 hair strands were cut from the posterior vertex, as close to the scalp as possible. The proximal 3 cm of hair, reflecting roughly the 3 months before the hair sample collection, were used to measure cortisol. Hair sample preparation has been described in detail [36,37]. In brief, a minimum of 15 mg of hair was weighed, cut into small pieces, and placed in a glass vial. Methanol was added to extract cortisol from the hair samples during overnight incubation (16 h) at 52°C. Afterward, the methanol was transferred into a clean glass vial and was evaporated under a nitrogen stream until completely dry. The samples were dissolved in Phosphate Buffered Saline (PBS: pH 8.0) and vortexed until thoroughly mixed prior to the analysis. The analysis was performed on an Agilent 1200 series G6410 Triple Quard instrument (Agilent, USA) equipped with an automatic sample injector, degasser, column thermostat, diode array detector, and an electrospray ionization source. The cortisol result was corrected for the individual hair weight of the sample, the amount of methanol used for extraction and the reconstitution volume, and the resulting HCC is reported inpg/mg. All samples with analyzing the LC-MS Spectrum were assayed in duplicate and the average used in analyses.The average intra-and inter-assay coefficients of variation of 4.71% and 8.75%, respectively.

### Saliva cortisol measurement

Changes in levels of salivary cortisol following the Trier Social Stress Test for Children (TSST-C) were obtained (in addition to the hair samples) and assessed to allow a comparison of different methodologies of cortisol assessment. The TSST-C [38] is used to elicit a stress response in the laboratory environment. This is a standard, laboratory stressor designed to elicit psychological stress and cortisol responses. The TSST-C was 15 min long and consisted of a 5-min speech preparation period, a videotaped 5-min public speaking task, and a 5-min mental arithmetic task, performed while facing two evaluative, non-responsive audience members. Subjects were instructed not to drink, eat, or vigorously exercise in the 2 hours preceding the visit. The sessions were conducted at 2:00 p.m. Upon entering the experimental room all subjects were allowed to have a rest. Here after the first saliva cortisol sample was taken (cort1). Subsequently, subjects were waiting for TSST-C, and this phase lasted for about 15min before which the next cortisol sample (cort 2) was taken. Then all subjects performed the TSST-C. After they completed the task subjects were asked to deliver the last saliva cortisol sample (cort3; about 15 min after cort2). Then saliva sample were continued collectting every 15-minute intervals (cort 4–9). A total of nine saliva samples were collected during the TSST-C; Participants rinsed their mouths with water immediately before passively drooling into a 5 ml tube for 30 seconds for each sample. The samples were transferred to a -70°Cultra low freezer until they were assayed using a highly sensitive enzyme immunoassay that was specifically designed for use with saliva (Cat. No. SLV-2930 DGR Instruments GmbH, Germany). The test has a range of sensitivity from 3.00 to 200.00 ng/ml, and average intra-and inter-assay coefficients of variation of less than 5% and 10%.

### Measures of internalizing symptoms

Depressive symptoms were measured by the Children’s Depression Inventory (CDI) designed by Kovacs [39], which contains 27 items that measure five dimensionsof symptoms: negative mood, interpersonal problems, ineffectiveness, anhedonia, andnegative self-esteem. Each item is rated on a 3-point scale from “not present” to“highest severity.” The CDI is used to assess depression symptoms of 7–17 year-olds.A continuous measure of anxiety symptom counts were provided in this study.The reliability and validity of the Chinese version of the Children’s Depression Inventory (CDI-C) were tested in a previous study, which reported a Cronbach’s alpha of 0.88, and an intra-class correlation coefficient of 0.89 [40].

Anxiety symptoms were measured by the Screen Scale for Child Anxiety Related Emotional Disorders (SCARED) [41]. The SCARED, which was designed by Birmaher and colleagues, is a 41-item questionnaire suitable for assessing anxiety symptoms in 9–18 year-olds. The SCARED measures five dimensions of symptoms: somatic/panic;generalized anxiety; separation anxiety; social phobia; and school phobia. Each item is rated on a 3-point scale from “not present” to “always present”. A continuous measure of anxiety symptom counts were provided in this study.The SCARED has been shown to have acceptable test-retest reliability (Pearson’s r = 0.57 to 0.61), internal consistency (Cronbach’s α = 0.43 to 0.89), and both high sensitivity and specificity to assess anxiety syndrome among Chinese children [42].

### Data analyses

Statistical analyses were performed using SPSS 16.0 for Windows (SPSS Inc., Chicago, IL, USA). The saliva cortisol area-under-the-curve-increase (AUCi) was calculated as a measure of cortisol dynamics [43]. This measure highlights changes over time from baseline, thus, providing a measure of person-specific changes in cortisol output over the course of the procedure, which is related to the sensitivity of the system [44]. As the data were not normally distributed, natural logarithmic transformations were performed on the cortisol values before the AUCi calculations. The levels of hair cortisol for the total sample were normally distributed after the log transformation. Univariate and multivariate liner regression coefficients were estimated for all indicators with hair cortisol and saliva cortisol data. To test whether this association between internalizing symptoms level and cortisol level had the gender difference, interaction terms between sex and internalizing symptoms were also added to analyse. Finally, these were then as linear multivariate regression models by sex, and we assessed the association between hair logcortisol level or saliva logAUCi and scores of internalizing symptoms, adjusted for other characteristics variables in the models. *P*<0.05 is used to define statistical significance.

## Results

Sociodemographic and anthropometric characteristics, internalizing symptoms, AUCi in saliva, and cortisol level in hair of all participants are shown in Table 1. Univariate liner regression on hair cortisol levels or saliva cortisol reactivity (AUCi) were presented for sex, age, BMI-Z, school-level, Tanner-stage, parental educational level, family income, and internalizing symptoms levels (Table 2). Hair cortisol level was higher in girls than in boys (*p* = 0.008). Lower family income was associated with higher hair cortisol levels (*p* = 0.013). Mother’s education level was positive associated to hair cortisol levels (*p* = 0.008). The scores of CDI were positive associated with cortisol level in hair, but the association was not significantly (*p* = 0.060). Multivariate liner regression analysis estimated for the indicators with hair cortisol or saliva cortisol logAUCi were presented in Table 2. The significant relative gender*CDI score interaction terms (*β* = -1.168, *p* = 0.012) indicated that the positive association between hair cortisol and depressive symptoms was stronger among boys, for whom being depression symptoms was less common. The scores of SCARED were not significantly related to hair cotisol level (*p* = 0.257) (Table 2). However, the significant relative sex* SCARED score interaction terms indicated that the negative association between hair cortisol and anxiety symptoms was stronger among girls (*β* = -1.129, *p* = 0.002). We also found a significant negative association of saliva cortisol AUCi with family income in univariate liner regression (*p* = 0.033). In multivariate liner regression, the significant relative sex* SCARED score interaction terms on saliva cortisol AUCi (*β* = -1.458, *p* = 0.021). No associations between saliva cortisol and CDI score were found with the linear regression model (Table 2).

**Table 1:** Characteristics of the boys (n = 46) and girls (n = 39).

Variable	Total	Boys	Girls
	Mean±SD	Mean±SD	Mean±SD
**Age**	11.40±0.33	11.41±0.35	11.39±0.31
**BMI**	21.26±4.07	22.20±4.43	20.15±3.34
**BMI-Z**	0.00±1.00	0.00±1.08	0.02±0.82
**SCARED score**	11.79±9.61	9.96±8.17	13.96±10.79
**CDI score**	8.89±3.91	9.88±4.59	7.72±2.49
**logAUCi (ng/ml saliva)**	-1.95±0.81	-0.28±7.42	-3.39±8.54
**logCortisol (pg/mg hair)**	1.07±0.39	0.97±0.36	1.19±0.39
	Median (range)	Median (range)	Median (range)
**Cortisol (pg/mg hair)**	12.02(1.23–91.70)	7.64(1.23–87.90)	17.76(1.66–91.70)
	Yes(n)	Yes(n)	Yes(n)
**School-level**			
1	42	22	20
2	43	24	19
**Puberty Stage**			
Stage I	26	14	12
Stage II	42	22	20
Stage III	17	10	7
Stage IV	0	0	0
Stage V	0	0	0
**Paternal education level**			
Junior high school or less	21	14	7
Senior high school	33	15	18
College or higher	31	17	14
**Maternal education level**			
Junior high school or less	27	18	9
Senior high school	36	16	20
College or higher	22	12	10
**Family income**			
Low	8	5	3
middle	59	30	29
high	18	11	7

BMI-Z score is defined as the number of standard deviation units from the mean or reference value.

AUCi: area-under-the-curve-increase of saliva cortisol reactivity.

CDI: Children’s Depression Inventory.

SCARED: Scale for Child Anxiety Related Emotional Disorders.

**Table 2:** Associations between different indicators and hair cortisol level or saliva cortisol reactivity.

Variable	logCortisol (pg/mg hair)		logAUCi(ng/ml saliva)	
	Univariate	Multivariate	Univariate	Multivariate
	*β* (SE)	*β* (SE)	*β* (SE)	*β* (SE)
Gender	0.286(0.081)*	1.242*(0.201)	−0.225(1.732)	0.064(4.893)
Age	−0.033(0.124)	−0.089(0.104)	0.052(2.284)	−0.198(2.839)
BMI-Z	0.193(0.004)	0.032(0.030)	0.051(0.913)	0.018(1.960)
School-level	−0.052(0.082)	−0.131(0.072)	0.180(0.139)	0.018(1.960)
Puberty Stage	0.047(0.058)	0.155(0.057)	0.009(1.765)	0.207(1.564)
Paternal education level	0.208(0.052)	0.154(0.050)	−0.034(1.154)	−0.032(1.267)
Maternal education level	0.280(0.052)**	0.054(0.048)	0.036(1.170)	−0.080(1.320)
Family Income	−0.273(0.073)*	−0.174(0.057)	−0.232(1.595)*	−0.231(1.564)*
Scores of CDI	0.248(0.009)*	0.807(0.021)	0.062(0.212)	−1.241(0.361)
Scores of SCARED	−0.56(0.114)	0.788(0.010)	−0.135(0.091)	1.107(0.313)
Gender×Scores of CDI		−1.168* (0.016)		0.294(0.562)
Gender×Scores of SCARED		−1.129**(0.007)		−1.458(0.192)**
R^2^		0.57		0.32

*P <0.05.

**P <0.01.

In Table 3, with linear regression analysis by sex, adjusted for parental education leval and family income, we found a significant association of hair logcortisol with the CDI score in boys (*p*<0.001). In girls, a significant association of both hair logcortisol (*p* = 0.006) and saliva cortisol logAUCi (*p* = 0.021) with the SCARED score.

**Table 3:** Association between measures of internalizing symptoms and hair cortisol or saliva cortisol reactivity by genders.

	Boys		Girls	
	logCortisol (pg/mg hair)	logAUCi (ng/ml saliva)	logCortisol (pg/mg hair)	logAUCi (ng/ml saliva)
	*β* (SE)	*β* (SE)	*β* (SE)	*β* (SE)
**Univariate Liner regression**				
Scores of Depressive Symptoms	0.553(0.010) **	0.052(0.244)	0.252(0.006)	−0.007(0.563)
Scores of Anxiety Symptoms	−0.135(0.025)	0.276(0.132)	−0.570(0.005)**	−0.427(0.118)**
**Multivariate Liner regression**				
Scores of Depressive Symptoms	−0.487(0.010) **	0.013(0.247)	Not adjusted	
Scores of Anxiety Symptoms	Not adjusted		−0.535(0.005) **	−0.413(0.125) *

Multivariate Liner regression analyses coefficients *β* after adjusting for parental education level and family income variables.

*P <0.05.

**P <0.01.

## Discussion

To our knowledge, this is one of the first studies to investigate the association between ratings of self-reported internalizing symptoms and HPA-axis activity, which were assessed by two different methodologies—long-term cortisol concentrations in the hair and cortisol reactivity to acute stress in the saliva of pre- and young adolescents. This study found a positive association between ratings of depressive symptoms and cumulative hair cortisol but not saliva cortisol activity among boys. Furthermore, this study found a negative association of anxiety symptoms levels with cumulative hair cortisol and acute cortisol reactivity in the saliva of girls.

This study revealed significant sex differences in the levels of hair cortisol, with girls having higher levels of hair cortisol than boys. Contradictory, several studies have found opposite results or no difference in sex. For example, a study in a large cohort of 2400 children (mean age 6 year) a higher level of hair cortisol was found in boys compared to girls [45], and another study on hair cortisol reference ranges in children and adoscents aged 4–18 years found no sex differences [46]. One explanation for this sex difference may lie in the different age ranges of the samples. In our study, the participants aged 10–12 years, who are in pre- or early puberty period. As there are sex differences in HPA regulation that appear at puberty [9,29], several studies have shown that adolescent girls have higher levels of free cortisol [32], upregulation of saliva cortisolactivity across the day [33], and a higher cortisol response upon awakening than boys [34]. The emergence of sex differences in reproductive hormones at puberty may play a role, as testosterone and other androgens have been shown to suppress CRH-stimulated HPA activity in men [47]. Studies exploring sex differences in levels of hair cortisol in pre- and young adolescents are extremely limited, although some non-human studies have found that female primates have higher levels of hair cortisol compared to males, beginning at puberty [33].

Importantly, the study was designed to investigate whether internalizing symptoms are associated with aberrant HPA-axis activity in pre- and early adolescents by examining cortisol concentrations in hair. This study found a positive association between rating of depressive symptoms and hair cortisol level only in boys. There is a growing research literature that suggests cortisol has differential associations with specific dimensions of depression in pre- adolescents, and that some depressive problems are related to HPA-axis dysregulation—forms of hyperactivity or hypoactivity, especially in boys [22,48]. For example, Dietrich et al. [48] who tested the association between day saliva cortisol and dimensions of depression in 2,049 10–12 year-old preadolescents, found a positive relationship between the saliva cortisol awakening response and depression only in boys. Fries et al. [49] suggested that the higher saliva cortisol awakening response provided an “energetic boost” to deal with the demands of the new day and to facilitate engagement with the social environment. However, research on hair cortisol concentrations in pre- and young adolescent samples has not been reported. In our study, this would initially suggest that higher hair cortisol concentrations were related to depressive symptoms during the period that hair segment grew, specifically in pre- and young adolescent boys. In contrast, parameters reflecting cortisol reactivity to the TSST-C (AUCi) were not significant, indicating there was no difference in cortisol reactivity to acute stress, per se, based on the self-reported depressive symptoms of boys. Thus, it is important to know this association was found solely for the measure of retrospective cortisol concertration in boys. Might boys who suffer from depressive symptoms be more vulnerable than girls to the neurotoxicity effects of persistently elevated cortisol concentration? If this is confirmed by future research, these findings highlight important differences in the information derived from different methods of cortisol assessment and they could suggest that depression may be associated with chronic hypercortisolism in pre- and young adolescent boys.

On the other hand, no association was found in girls between levels of depressive symptoms and hair cortisol or salivary cortisol reactivity. This finding stands in contrast to previous research suggesting there is an association between depression symptoms or disorders and dysregulation of the HPA axis, especially among adolescent girls [9,14]. Given previous research showed that girls had a greater rate of depressive symptoms, which appear to emerge during adolescence [19–21], a growing body of theory and research supports the role of pubertal processes in the emergence of sex differences in depression [50,51]. The possibility that the HPA axis is maximally sensitive to stress experiences during adolescence has implications for psychopathology, especially for females [22]. Previous findings showed the rate of depression in preadolescent adoscents is approximately equal to or slightly higher in boys than girls [20], and that the higher rate of depressive symptoms and depressive disorders in girls appears to emerge around age 13–14 years [21,52]. In our study, there were also no significant associations between higher levels of depressive symptoms and abnormal HPA axis activity when both long-term cortisol concentration in hair and acute-term cortisol reactivities are assessed concurrently in pre- and young adolescent girls.

This study found a negative association between ratings of anxiety symptoms and cumulative hair cortisol as well as saliva cortisol reactivity in girls. Consistent with the results of a recent review Staufenbiel [23] demonstrating that lower hair cortisol concentrations were related to anxiety (generalized anxiety disorder and panic disorder) among adults, early adolescent girls with higher ratings of anxiety in our study also showed lower cortisol secretions in hair. Moreover, decreased saliva cortisol reactivity was associated with higher SCARED scores, suggesting that individuals with anxiety symptoms presented blunted HPA-axis activity under an acute stress condition. The model of a hypoactive HPA axis supports the theory developed by Gunnar and Vazquez [53] that stressful experiences early in life may elicit frequent elevations in cortisol levels and gradually result in an attenuation of cortisol secretion, i.e., HPA-axis hypoactivity due to increasing chronicity compensatory mechanisms. In addition, no significant association was found between hair cortisol concentrations and salivary measures of acute cortisol reactivity in either gender in current study. In contrast, van Holland et al. [54] found that short-term cortisol excretion in saliva and long-term cortisol excretion in hair were significantly associated. Possible explanations for the inconsistent finding may be that participants in van Holland’s study were instructed to collect 6 samples at prescribed times each, and saliva samples were collected for 3 days. However, acute cortisol reactivity in the current study only represents the body’s response to perceived acute stress and the ability of the HPA axis to regulate these responses. It is conceivable that the extent of such HPA-axis regulation followed by acute stress might not be related to long-term cortisol secretion in hair under normal conditions. After all, hair cortisol concentrations reflect all cortisol exposure over a given period in an integrated way. Another explanation could be that the relatively small sample size reduced the ability to detect significant relationships. Of note, previous investigations suggested the higher levels of hair cortisol in children and adolescents in family with low income or low socioeconomic status [45,55]. Such covariates and confounders in this study were taken into account. We observed that family income were significantly negative related to levels of hair cortisol as well as saliva cortisol reactivity in this study.

The study findings are limited by the small, racially homogeneous and urban samples, which preclude their generalizability to more diverse populations. Another limitation of this study is that exceptfor morphological indices, all other information was selfreported by adolescents. The study used internalizing symptoms screening scales rather than clinical diagnostic criteria toevaluate depressive symptoms and anxiety. Finally, it must be emphasized that hair cortisol analysis is a novel methodology with many details that still require close examination. Thus, one cannot exclude the possibility that unknown factors (e.g., hair wash frequency, group differences incortisol incorporation into hair or cortisol metabolism rates) may have influenced the present findings.

## Conclusions

In summary, this study is a first step in research on the association between self-reported internalizing symptoms and hair cortisol concentrations and cortisol reactivity in saliva in pre- and young adolescent samples aged 10–12 year-old. This study found a positive association between ratings of current depressive symptoms and cumulative hair cortisol levels only in boys. Furthermore, higher ratings of anxiety symptoms were associated with lower long-term hair cortisol excretion and lower saliva cortisol reactivity under acute stress among girls aged 10–12 years. This provides the first evidence for the notion that depressive symptoms in boys are associated with long-term cortisol concertration, whereas anxiety symptoms in girls are associated with HPA-axis hypoactivity, when long-term cortisol concentration and acute cortisol reactivity are assessed concurrently.

## Supporting information

S1 File Dataset. (SAV)

## References

[1] Goodwin RD, Fergusson DM, Horwood LJ. Early anxious/withdrawn behaviours predict later internalising disorders. J Child Psychol Psychiatry. 2004;45(4):874–83. doi: 10.1111/j.1469-7610.2004.00279.x 15056317 10.1111/j.1469-7610.2004.00279.x

[2] Brady EU, Kendall PC. Comorbidity of anxiety and depression in children and adolescents. Psychological bulletin. 1992;111(2):244–55. 1557475 10.1037/0033-2909.111.2.244

[3] Vreeburg SA, Hoogendijk WJ, van Pelt J, DeRijk RH, Verhagen JC, van Dyck R, et al Major depressive disorder and hypothalamic-pituitary-adrenal axis activity: results from a large cohort study. Archives of general psychiatry. 2009;66(6):617–26. doi: 10.1001/archgenpsychiatry.2009.50 19487626 10.1001/archgenpsychiatry.2009.50

[4] Stetler C, Miller GE. Depression and hypothalamic-pituitary-adrenal activation: a quantitative summary of four decades of research. Psychosomatic medicine. 2011;73(2):114–26. doi: 10.1097/PSY.0b013e31820ad12b 21257974 10.1097/PSY.0b013e31820ad12b

[5] Ruttle PL, Shirtcliff EA, Serbin LA, Fisher DB, Stack DM, Schwartzman AE. Disentangling psychobiological mechanisms underlying internalizing and externalizing behaviors in youth: longitudinal and concurrent associations with cortisol. Hormones and behavior. 2011;59(1):123–32. doi: 10.1016/j.yhbeh.2010.10.015 21056565 10.1016/j.yhbeh.2010.10.015 PMC3066166

[6] Rao U, Hammen C, Ortiz LR, Chen LA, Poland RE. Effects of early and recent adverse experiences on adrenal response to psychosocial stress in depressed adolescents. Biological psychiatry. 2008;64(6):521–6. doi: 10.1016/j.biopsych.2008.05.012 18597740 10.1016/j.biopsych.2008.05.012 PMC2559463

[7] Stroud LR, Papandonatos GD, Williamson DE, Dahl RE. Sex differences in cortisol response to corticotropin releasing hormone challenge over puberty: Pittsburgh Pediatric Neurobehavioral Studies. Psychoneuroendocrinology. 2011;36(8):1226–38. doi: 10.1016/j.psyneuen.2011.02.017 21489699 10.1016/j.psyneuen.2011.02.017 PMC3270708

[8] Adam EK, Doane LD, Zinbarg RE, Mineka S, Craske MG, Griffith JW. Prospective prediction of major depressive disorder from cortisol awakening responses in adolescence. Psychoneuroendocrinology. 2010;35(6):921–31. doi: 10.1016/j.psyneuen.2009.12.007 20079576 10.1016/j.psyneuen.2009.12.007 PMC2875344

[9] Van den Bergh BR, Van Calster B. Diurnal cortisol profiles and evening cortisol in post-pubertal adolescents scoring high on the Children's Depression Inventory. Psychoneuroendocrinology.2009;34(5):791–4. doi: 10.1016/j.psyneuen.2008.12.008 19171435 10.1016/j.psyneuen.2008.12.008

[10] Feder A, Coplan JD, Goetz RR, Mathew SJ, Pine DS, Dahl RE, et al Twenty-four-hour cortisol secretion patterns in prepubertal children with anxiety or depressive disorders. Biological psychiatry.2004;56(3):198–204. doi: 10.1016/j.biopsych.2004.05.005 15271589 10.1016/j.biopsych.2004.05.005

[11] Kryski KR, Smith HJ, Sheikh HI, Singh SM, Hayden EP. HPA axis reactivity in early childhood: associations with symptoms and moderation by sex. Psychoneuroendocrinology.2013;38(10):2327–36. doi: 10.1016/j.psyneuen.2013.05.002 23764193 10.1016/j.psyneuen.2013.05.002

[12] Badanes LS, Watamura SE, Hankin BL. Hypocortisolism as a potential marker of allostatic load in children: associations with family risk and internalizing disorders. Development and psychopathology. 2011;23(3):881–96. doi: 10.1017/S095457941100037X 21756439 10.1017/S095457941100037X PMC4072203

[13] Hankin BL, Badanes LS, Abela JR, Watamura SE. Hypothalamic-pituitary-adrenal axis dysregulation in dysphoric children and adolescents: cortisol reactivity to psychosocial stress from preschool through middle adolescence. Biological psychiatry. 2010;68(5):484–90. doi: 10.1016/j.biopsych.2010.04.004 20497900 10.1016/j.biopsych.2010.04.004 PMC2921478

[14] Keenan K, Hipwell A, Babinski D, Bortner J, Henneberger A, Hinze A, et al Examining the developmental interface of cortisol and depression symptoms in young adolescent girls. Psychoneuroendocrinology. 2013;38(10):2291–9. doi: 10.1016/j.psyneuen.2013.04.017 23726646 10.1016/j.psyneuen.2013.04.017 PMC3776001

[15] Kagan J, Reznick JS, Snidman N. Biological bases of childhood shyness. Science. 1988;240(4849):167–71. 3353713 10.1126/science.3353713

[16] Greaves-Lord K, Huizink AC, Oldehinkel AJ, Ormel J, Verhulst FC, Ferdinand RF. Baseline cortisol measures and developmental pathways of anxiety in early adolescence. Acta psychiatrica Scandinavica. 2009;120(3):178–86. doi: 10.1111/j.1600-0447.2009.01402.x 19485962 10.1111/j.1600-0447.2009.01402.x

[17] Bosch NM, Riese H, Dietrich A, Ormel J, Verhulst FC, Oldehinkel AJ. Preadolescents' somatic and cognitive-affective depressive symptoms are differentially related to cardiac autonomic function and cortisol: the TRAILS study. Psychosomatic Medicine. 2009;71(9):944–50. doi: 10.1097/PSY.0b013e3181bc756b 19834052 10.1097/PSY.0b013e3181bc756b

[18] Sondeijker FE, Ferdinand RF, Oldehinkel AJ, Tiemeier H, Ormel J, Verhulst FC. HPA‐axis activity as a predictor of future disruptive behaviors in young adolescents. Psychophysiology. 2008;45(3):398–404. doi: 10.1111/j.1469-8986.2008.00639.x 18312496 10.1111/j.1469-8986.2008.00639.x

[19] Andrade L, Caraveo-Anduaga JJ, Berglund P, Bijl RV, De Graaf R, Vollebergh W, et al The epidemiology of major depressive episodes: results from the International Consortium of Psychiatric Epidemiology (ICPE) Surveys. International journal of methods in psychiatric research. 2003;12(1):3–21. 12830306 10.1002/mpr.138 PMC6878531

[20] Kessler RC, Avenevoli S, Ries Merikangas K. Mood disorders in children and adolescents: an epidemiologic perspective. Biological psychiatry. 2001;49(12):1002–14. 11430842 10.1016/s0006-3223(01)01129-5

[21] Kessler RC, Berglund P, Demler O, Jin R, Merikangas KR, Walters EE. Lifetime prevalence and age-of-onset distributions of DSM-IV disorders in the National Comorbidity Survey Replication. Archives of general psychiatry. 2005;62(6):593–602. doi: 10.1001/archpsyc.62.6.593 15939837 10.1001/archpsyc.62.6.593

[22] Keenan K, Hipwell AE. Preadolescent clues to understanding depression in girls. Clin Child Fam Psychol Rev. 2005;8(2):89–105. 15984082 10.1007/s10567-005-4750-3 PMC2848388

[23] Wennig R. Potential problems with the interpretation of hair analysis results. Forensic science international. 2000;107(1–3):5–12. 10689559 10.1016/s0379-0738(99)00146-2

[24] Staufenbiel SM, Penninx BW, Spijker AT, Elzinga BM, van Rossum EF. Hair cortisol, stress exposure, and mental health in humans: a systematic review. Psychoneuroendocrinology.2013;38(8):122035 doi: 10.1016/j.psyneuen.2012.11.015 23253896 10.1016/j.psyneuen.2012.11.015

[25] Gerber M, Kalak N, Elliot C, Holsboer-Trachsler E, Puhse U, Brand S. Both hair cortisol levels and perceived stress predict increased symptoms of depression: an exploratory study in young adults. Neuropsychobiology. 2013;68(2):100–9. doi: 10.1159/000351735 23881183 10.1159/000351735

[26] Herane Vives A, De Angel V, Papadopoulos A, Strawbridge R, Wise T, Young AH, et al The relationship between cortisol, stress and psychiatric illness: New insights using hair analysis. J Psychiatr Res. 2015;70:38–49. doi: 10.1016/j.jpsychires.2015.08.007 26424422 10.1016/j.jpsychires.2015.08.007

[27] Faresjo A, Theodorsson E, Chatziarzenis M, Sapouna V, Claesson HP, Koppner J, et al Higher perceived stress but lower cortisol levels found among young Greek adults living in a stressful social environment in comparison with Swedish young adults. PLoS One. 2013;8(9):e73828 doi: 10.1371/journal.pone.0073828 24066077 10.1371/journal.pone.0073828 PMC3774738

[28] Steudte S, Stalder T, Dettenborn L, Klumbies E, Foley P, Beesdo-Baum K, et al Decreased hair cortisol concentrations in generalised anxiety disorder. Psychiatry Res. 2011;186(2–3):310–4. doi: 10.1016/j.psychres.2010.09.002 20889215 10.1016/j.psychres.2010.09.002

[29] McCormick CM, Mathews IZ. HPA function in adolescence: role of sex hormones in its regulation and the enduring consequences of exposure to stressors. Pharmacology, biochemistry, and behavior. 2007;86(2):220–33. doi: 10.1016/j.pbb.2006.07.012 16901532 10.1016/j.pbb.2006.07.012

[30] Lupien SJ, McEwen BS, Gunnar MR, Heim C. Effects of stress throughout the lifespan on the brain, behaviour and cognition. Nat Rev Neurosci. 2009;10(6):434–45. doi: 10.1038/nrn2639 19401723 10.1038/nrn2639

[31] Sondeijker FE, Ferdinand RF, Oldehinkel AJ, Veenstra R, Tiemeier H, Ormel J, et al Disruptive behaviors and HPA-axis activity in young adolescent boys and girls from the general population. J Psychiatr Res. 2007;41(7):570–8. doi: 10.1016/j.jpsychires.2006.04.002 16730747 10.1016/j.jpsychires.2006.04.002

[32] Goodyer IM, Bacon A, Ban M, Croudace T, Herbert J. Serotonin transporter genotype, morning cortisol and subsequent depression in adolescents. The British journal of psychiatry the journal of mental science. 2009;195(1):39–45. doi: 10.1192/bjp.bp.108.054775 19567894 10.1192/bjp.bp.108.054775 PMC2802528

[33] Shirtcliff EA, Allison AL, Armstrong JM, Slattery MJ, Kalin NH, Essex MJ. Longitudinal stability and developmental properties of salivary cortisol levels and circadian rhythms from childhood to adolescence. Developmental psychobiology. 2012;54(5):493–502. doi: 10.1002/dev.20607 21953537 10.1002/dev.20607 PMC3270212

[34] Platje E, Vermeiren RR, Branje SJ, Doreleijers TA, Meeus WH, Koot HM, et al Long-term stability of the cortisol awakening response over adolescence. Psychoneuroendocrinology. 2013;38(2):271–80. doi: 10.1016/j.psyneuen.2012.06.007 22776421 10.1016/j.psyneuen.2012.06.007

[35] Laudenslager ML, Jorgensen MJ, Fairbanks LA. Developmental patterns of hair cortisol in male and female nonhuman primates: lower hair cortisol levels in vervet males emerge at puberty. Psychoneuroendocrinology. 2012;37(10):1736–9. doi: 10.1016/j.psyneuen.2012.03.015 22497987 10.1016/j.psyneuen.2012.03.015 PMC3398229

[36] Manenschijn L, Spijker AT, Koper JW, Jetten AM, Giltay EJ, Haffmans J, et al Long-term cortisol in bipolar disorder: associations with age of onset and psychiatric co-morbidity. Psychoneuroendocrinology. 2012;37(12):1960–8. doi: 10.1016/j.psyneuen.2012.04.010 22634056 10.1016/j.psyneuen.2012.04.010

[37] Sauve B, Koren G, Walsh G, Tokmakejian S, Van Uum SH. Measurement of cortisol in human hair as a biomarker of systemic exposure. Clinical and investigative medicine Medecine clinique et experimentale. 2007;30(5):E183–91. 17892760 10.25011/cim.v30i5.2894

[38] Dickerson SS, Kemeny ME. Acute stressors and cortisol responses: a theoretical integration and synthesis of laboratory research. Psychological bulletin. 2004;130(3):355–91. doi: 10.1037/0033-2909.130.3.355 15122924 10.1037/0033-2909.130.3.355

[39] Kovacs M Children’s Depression Inventory (CDI). Manual Toronto, ON:Multi Health Systems, 1992: 15–25.

[40] Wu WF, Lu YB, Tan FR, Yao SJ. Reliability and Validity of the Chinese Versionof Children’s Depression Inventory. Chinese Mental Health Journal. 2010; 24: 775–9. (In Chinese with English abstract).

[41] Birmaher B, Brent DA, Chiappetta L, Bridge J, Monga S, Baugher M. Psychometric properties of the Screen for Child Anxiety Related Emotional Disorders (SCARED): a replication study. Journal of the American Academy of Child and Adolescent Psychiatry. 1999;38(10):1230–6. doi: 10.1097/00004583-199910000-00011 10517055 10.1097/00004583-199910000-00011

[42] Wang K, Su LY, Zhu Y, Zhai J, Yang ZW, Zhang JS. Norms of the Screen for Child Anxiety Related Emotional Disorders in Chinese Urban Children. Chinese Journal of Clinical Psychology. 2002; 10:270–2. (In Chinese with English abstract)

[43] Fekedulegn DB, Andrew ME, Burchfiel CM, Violanti JM, Hartley TA, Charles LE, et al Area under the curve and other summary indicators of repeated waking cortisol measurements. Psychosom Med. 2007;69(7):651–9. doi: 10.1097/PSY.0b013e31814c405c 17766693 10.1097/PSY.0b013e31814c405c

[44] Granger DA, Fortunato CK, Beltzer EK, Virag M, Bright MA, Out D. Focus on methodology: salivary bioscience and research on adolescence: an integrated perspective. Journal of adolescence. 2012;35(4):1081–95. doi: 10.1016/j.adolescence.2012.01.005 22401843 10.1016/j.adolescence.2012.01.005

[45] Rippe RC, Noppe G, Windhorst DA, Tiemeier H, van Rossum EF, Jaddoe VW, et al Splitting hair for cortisol? Associations of socio-economic status, ethnicity, hair color, gender and other child characteristics with hair cortisol and cortisone. Psychoneuroendocrinology. 2016;66:56–64. doi: 10.1016/j.psyneuen.2015.12.016 26773401 10.1016/j.psyneuen.2015.12.016

[46] Noppe G, Van Rossum EF, Koper JW, Manenschijn L, Bruining GJ, de Rijke YB, et al Validation and reference ranges of hair cortisol measurement in healthy children. Horm Res Paediatr. 2014;82(2):97–102. doi: 10.1159/000362519 25115629 10.1159/000362519

[47] Rubinow DR, Roca CA, Schmidt PJ, Danaceau MA, Putnam K, Cizza G, et al Testosterone suppression of CRH-stimulated cortisol in men. Neuropsychopharmacology official publication of the American College of Neuropsychopharmacology. 2005;30(10):1906–12. doi: 10.1038/sj.npp.1300742 15841103 10.1038/sj.npp.1300742 PMC1470424

[48] Dietrich A, Ormel J, Buitelaar JK, Verhulst FC, Hoekstra PJ, Hartman CA. Cortisol in the morning and dimensions of anxiety, depression, and aggression in children from a general population and clinic-referred cohort: An integrated analysis. The TRAILS study. Psychoneuroendocrinology. 2013;38(8):1281–98. doi: 10.1016/j.psyneuen.2012.11.013 23237815 10.1016/j.psyneuen.2012.11.013

[49] Fries E, Dettenborn L, Kirschbaum C. The cortisol awakening response (CAR): facts and future directions. Int J Psychophysiol. 2009;72(1):67–73. doi: 10.1016/j.ijpsycho.2008.03.014 18854200 10.1016/j.ijpsycho.2008.03.014

[50] Parker GB, Brotchie HL. From diathesis to dimorphism: the biology of gender differences in depression. J Nerv Ment Dis. 2004;192(3):210–6. 15091302 10.1097/01.nmd.0000116464.60500.63

[51] Born L, Shea A, Steiner M. The roots of depression in adolescent girls: is menarche the key? Curr Psychiatry Rep. 2002;4(6):449–60. 12441025 10.1007/s11920-002-0073-y

[52] Twenge JM, Nolen-Hoeksema S. Age, gender, race, socioeconomic status, and birth cohort differences on the children's depression inventory: a meta-analysis. J Abnorm Psychol. 2002;111(4):578–588. 12428771 10.1037//0021-843x.111.4.578

[53] Gunnar MR, Vazquez DM. Low cortisol and a flattening of expected daytime rhythm: potential indices of risk in human development. Dev Psychopathol. 2001;13(3):515–38. 11523846 10.1017/s0954579401003066

[54] van Holland BJ, Frings-Dresen MH, Sluiter JK. Measuring short-term and long-term physiological stress effects by cortisol reactivity in saliva and hair. Int Arch Occup Environ Health. 2012;85(8):849–52. doi: 10.1007/s00420-011-0727-3 22183048 10.1007/s00420-011-0727-3 PMC3480579

[55] Vliegenthart J, Noppe G, van Rossum EF, Koper JW, Raat H, van den Akker EL. Socioeconomic status in children is associated with hair cortisol levels as a biological measure of chronic stress. Psychoneuroendocrinology. 2016;65:9–14. doi: 10.1016/j.psyneuen.2015.11.022 26708067 10.1016/j.psyneuen.2015.11.022

